# Supplementary material for: Structure-based mechanism of RyR channel operation by calcium and magnesium ions
Source: PLoS Comput Biol. 2025 Apr 29;21(4):e1012950. doi: 10.1371/journal.pcbi.1012950 (PMC12119028; doi:10.1371/journal.pcbi.1012950)
Supplement: S1 Table — This table complements Table 9. (PDF) [file pcbi.1012950.s004.pdf]

S1 Table. Fractional occurrence of branches in the inhibition network pathways.

	Inhibition branches	Fraction of paths (%)								
		RyR1					RyR2			
		C	P	O	$O^{\dagger}$	$I^{\dagger}$	C	$C^{\dagger}$	$O^{\dagger}$	O
		7k0t	7tzc	7m6l	7tdh	7tdg	7vmm	7ua5	7ua9	7vmp
	Branches of the intra-monomeric pathway									
I1	INH-EF-U-K4214-S6-GATE		42	72	60		100	63		100
I2	INH-EF-U-T4979-S6-GATE								18	
I3	INH-EF-U-L4985, I4218-S6-GATE								74	
I4	INH-EF-U-K4211- K4821,T4822-S6-GATE		53			48		24		
I5	INH-EF-U-T4979-S4828, S4829-S6-GATE				27					
I6	INH-EF-U-K4211-K4821, S4828, S4829-S6-GATE	100			4					
	Subtotal	100	95	72	91	48	100	$88^{\#}$	92	100
	Branches of the inter-monomeric pathway									
I7	INH-EF-E4075/R4736*-U*-S6*-GATE			14	9					
I8	INH-EF-S4099/I4731*-U*-S6*-GATE			14						
I9	INH-EF-K4101/D4730*-U*-S6*-GATE		5							
I10	INH-EF-K4101/I4731*-U*- R4824*, S4828*, S4829*-S6*-GATE								8	
I11	INH-EF-E4075/R4736*- R4824*, S4828*, S4829*-S6*-GATE					40				
I12	INH-EF-K4101/D4730*- R4824*, S4828*, S4829*-S6*-GATE					12				
	Subtotal	0	5	28	9	52	0	0	8	0
	TOTAL	100	100	100	100	100	100	$88^{\#}$	100	100

INH and GATE are defined in Table 2 of the main text. The font color of a residue indicates its partaking: blue font – in the ATP binding site; brown font – in the S45 segment; green font – in the EF-hand domain; orange font – in the S23* loop.

† marks a pair of RyR1 and RyR2 structures obtained in the same experiment.

S1 Table complements Table 4.
