# Supplementary material for: Structure-based mechanism of RyR channel operation by calcium and magnesium ions
Source: PLoS Comput Biol. 2025 Apr 29;21(4):e1012950. doi: 10.1371/journal.pcbi.1012950 (PMC12119028; doi:10.1371/journal.pcbi.1012950)
Supplement: S2 Table — This table complements Table 6. (PDF) [file pcbi.1012950.s005.pdf]

S2 Table. Fractional occurrence of branches in the activation network pathways.

Activation pathways		Fraction of paths (%)								
		RyR1					RyR2			
		C	P	O	O†	I†	C	C†	O†	O
		7k0t	7tzc	7m6l	7tdh	7tdg	7vmm	7ua5	7ua9	7vmp
	ATP-site branches									
A1	ACT-CTD-U-K4214-S6-GATE			100		20	100	80		18
A2	ACT-(CD)-CTD-U-K4957-S6-GATE	60	38						7	21
A3	ACT-CTD-U-I4218-S6-GATE	40								
A4	ACT-CTD-U-F4959-U-S6-GATE				69					
A5	ACT-CTD-W5011-U-K4957-S6-GATE									25
A6	ACT-CTD-U-K4211-K4821,T4822-S6-GATE					77		20		
	Subtotal	60	38	100	69	97	100	100	7	64
	S45 branches									
A7	ACT-CTD-U-S4828,S4829-S6-GATE				14					
A8	ACT-CTD-U-R4824,T4825,I4826-S6-GATE								93	
	Subtotal	0	0	0	14	0	0	0	93	0
	CFF-site branch									
A9	ACT-CTD-W5011-U-S6-GATE		61		17					36
	Subtotal		61		17					36
	TOTAL	100	99	100	100	97	100	100	100	100

ACT and GATE are defined in Table 2 of the main text. The font color of a residue indicates its partaking: blue font – in the ATP binding site; brown font – in the S45 segment; cyan font – in the caffeine binding site. † marks a pair of RyR1 and RyR2 structures obtained in the same experiment.

S2 Table complements Table 5.
