# Supplementary material for: Structure-based mechanism of RyR channel operation by calcium and magnesium ions
Source: PLoS Comput Biol. 2025 Apr 29;21(4):e1012950. doi: 10.1371/journal.pcbi.1012950 (PMC12119028; doi:10.1371/journal.pcbi.1012950)
Supplement: S3 Table — This table complements Table 7. (PDF) [file pcbi.1012950.s006.pdf]

S3 Table. **Interaction between the activation network and inhibition network pathways.**

		ACT-CTD-EF-U-K4214-S6-G	ACT-CTD-EF-U-K4957-S6-G	ACT-CTD-EF-U-I4218-S6-G	ACT-CTD-EF-U-F4959-EF-U-S6-G	ACT-CTD-W5011-EF-U-K4957-S6-G	ACT-CTD-EF-U-K4211-K4821,T4822-S6-G	ACT-CTD-EF-U-S4828,S4829-S6-G	ACT-CTD-EF-U-R4824,T4825,I4826-S6-G	ACT-CTD-W5011-EF-U-S6-G
		A1	A2	A3	A4	A5	A6	A7	A8	A9
I1	INH-EF-U-K4214-S6-G	7m6l$ 7vmm$ 7ua5$ 7vmp$	7tzc 7vmp		7tdh	7vmp				
I2	INH-EF-U-T4979-S6-G		7ua9							
I3	INH-EF-U-L4985, I4218-S6-G		7ua9							
I4	INH-EF-U-K4211- K4821,T4822-S6-G	7tdg	7tzc				7tdg$ 7ua5$			
I5	INH-EF-U-T4979-S4828,S4829-S6-G				7tdh					
I6	INH-EF-U-K4211-K4821,S4828,S4829-S6-G		7k0t	7k0t	7tdh			7tdh$		
I7	INH-E4075/R4736*-U*-S6*-G*									
I8	INH-S4099/I4731*-U*-S6*-G*									
I9	INH-K4101/D4730*-U*-S6*-G*									
I10	INH-K4101/D4730*-R4824*, S4828*, S4829*-S6*-G*						7tdg#			
I11	INH-E4075/R4736*-R4824*,S4828*,S4829*-S6*-G*						7tdg#			
I12	INH-K4101/I4731*-U*-R4824*,S4828*,S4829*-S6*-G*								7ua9$#	

ACT, INH, and GATE are defined in Table 2 of the main text. The font color of a residue indicates its partaking: blue font – in the ATP binding site; cyan font – in the caffeine binding site; brown font – in the S45 segment; green font – in the EF-hand domain; orange font – in the S23* loop.

marks the interaction of the inter-monomeric inhibition pathway with the activation pathway of the anticlockwise-neighboring monomer.

$ marks structures where a residue of the ATP-binding site, or the S45 segment, participates in both the inhibition and activation network.

S3 Table complements Table 6.
