# Supplementary material for: Structure-based mechanism of RyR channel operation by calcium and magnesium ions
Source: PLoS Comput Biol. 2025 Apr 29;21(4):e1012950. doi: 10.1371/journal.pcbi.1012950 (PMC12119028; doi:10.1371/journal.pcbi.1012950)
Supplement: Attachment — Submitted filename: RC-2024-02632_full-revision_responses_PLoS-CB.docx [file pcbi.1012950.s015.docx]

**Manuscript number:** RC-2024-02632

**Corresponding author(s):** Alexandra Zahradníková, Ivan Zahradník

## 1. General Statements [optional]

*We thank the reviewers for their thorough and positive evaluation of the manuscript.*

## 2. Point-by-point description of the revisions

We revised the manuscript following the suggestions of the reviewers to make the article more concise and comprehensible to a wider audience. Specifically, we rearranged Section 5, rewrote the difficult-to-understand sections 5 and 6, and removed unnecessary or overlapping text in Introduction and Discussion. We added two sentences to the section Concluding Remarks on lines 982-983 (“This approach may find use in deciphering the allosteric mechanisms of ion channels in general.”) and 990-991 (Modulation of the inactivation pathways may be a prospective target for pharmacological interventions in relevant diseases of skeletal and cardiac muscles.). We have also addressed the specific points raised by the reviewers. To comply with PloS Computational Biology author instructions we have shortened the Abstract to 214 words.

The responses to individual points are detailed below.

Reviewer 1:

The reviewer did not ask for any changes to the manuscript.

*We thank the reviewer for the positive evaluation of the manuscript.*

Reviewer 2:

1/ Title: Structure-based mechanism of RyR channel operation by calcium and magnesium ions

The authors may consider using an alternative term instead of "operation".

*Thank you for the suggestion. We considered and discussed the term “RyR channel operation” very thoroughly with several colleagues, including native English speakers, and we found it to represent the complex RyR behavior in situ and in experiments most exactly. Alternative terms such as “control” suggest a one-way deterministic action from the ion binding to the protein state, which is not the case. The terms such as “modulation” implicate the presence of a higher RyR state-governing principle, such as phosphorylation, nitrosylation, binding of auxiliary proteins, etc.*

2/ Abstract: Please spell out CFF and MWC theorem.

*Thank you for the proposal. CFF was changed to* ***caffeine****; MWC was changed to* ***Monod-Wyman-Changeaux***

3/ Line 87-88: "In striated muscle cells, RyR channels cluster at discrete sites of sarcoplasmic reticulum attached to the sarcolemma where electrical excitation triggers transient calcium release by activation of RyRs."

There is no attachment between sarcoplasmic reticulum and sarcolemma, please rewrite.

*We respectfully disagree, since there is strong evidence for the existence of discrete contact sites between the sarcolemma and sarcoplasmic reticulum both at triads of skeletal muscle (Rossi et al., 2019) and at dyads of cardiac muscle (Mackrill, 2022), at which both membranes are firmly attached.*

*However, to avoid potential misunderstanding, we changed the sentence to “In striated muscle cells, RyR channels cluster at* ***the*** *discrete sites of sarcoplasmic reticulum attached to the sarcolemma* ***in triads or dyads****, where electrical excitation triggers transient calcium release by activation of RyRs” (lines 87-88).*

4/ Lines 104-107: "Recently, mathematical modeling of the cardiac calcium release site (Iaparov et al., 2022) confirmed that Mg2+ ions could at the same time act as the negative competitor at the calcium activation site and as an inhibitor at the inhibition site. Unfortunately, the structural counterpart of RyR inactivation, an inhibitory binding site for divalent ions, has not been located yet in RyR structures."

Note that the exact structural counterpart exists (Nayak et al., 2022, 2024), where Ca and Mg were found both at the activation and inhibition sites. The paragraph should be updated accordingly.

*We respectfully disagree. In the cited works of Nayak et al. (2022; 2024) it was shown that Ca and Mg ions bind firmly at the activation site. Both atoms were also observed at the ACP molecule bound at the ATP binding site. However, they were not observed at the divalent ion-binding inhibition site, which is distinct from the ATP binding site and resides in the loops of the EF-hand region.*

*However, to clarify the meaning of the disputed sentence, we have changed it to: “Although binding of* ***Ca^2+^ or Mg^2+^*** *to an inhibitory binding site has not been observed yet in RyR structures,* ***a consensus is emerging that the EF-hand loops constitute this site (Gomez et al., 2016; Zheng and Wen, 2020; Nayak et al., 2024; Chirasani et al., 2024 )****” (lines 108-110).*

5/ Lines 108-110: The activation of RyR by agonists was shown to be accompanied by a conformational change around the Ca2+ binding site that leads to a decrease in the free energy and to a concomitant increase of the Ca2+ binding affinity and a population shift between the closed and open conformations (Dashti et al., 2020).

Please clarify to what state does the "decrease in free energy" refer, to the open or to the closed state?

*Thank you for the proposal. The text was changed to: “The activation of RyR by agonists was shown to be accompanied by a conformational change around the Ca^2+^ binding site that leads to a decrease in the free energy* ***of the open state*** *and concomitantly to an increase of the Ca^2+^ binding affinity of the activation site. As a result, the occurrence probability of a RyR state/conformation* *shifts* ***from the closed toward the open*** *(Dashti et al., 2020)” (lines 111-114).*

6/ Figure 2: please indicate if distances were measured between the C-alphas or side chains.

*Thank you for the proposal. The figure legend was modified to “Distances D1 between* ***the Cα atoms of E4075 and*** *R4736* or equivalent. Right - Distances D2 between* ***the Cα atoms of K4101 and*** *D4730* or equivalent.”*

7/ Line 353-357: "These data suggest that interactions between the basic arginine residue R4736* and the acidic residues at the start of the initial helix E of the EF1-hand are specific for Ca2+-dependent inactivation in RyR1, whereas the interactions between the lysine K4101 that immediately follows the F helix of EF1 and the middle of the S23* loop (corresponding to D4730* and I4731* in RyR1) may play a part in the inactivation of both RyR1 and RyR2 isoforms.

Sentence is unclear; please rewrite. Overall, the entire section "Spatial interactions between the EF-hand and S23* regions" should be simplified and shortened.

*Thank you for the proposal. The text was changed to: "These data suggest that interactions between the basic arginine residue R4736* and the acidic residues* ***E4075 and D4079*** *are specific for Ca^2+^-dependent inactivation in RyR1, whereas the interactions between the lysine K4101* ***and the residues D4730* and I4731* (rRyR1 notation)*** *may play a part in the inactivation of both RyR1 and RyR2 isoforms.” (lines 334-337).*

*We did not find a way how to make the whole section simpler and shorter at the same time without losing clarity.*

8/ Lines 246-249 and Table 1. "all structures corresponding to rRyR1 residues 4063-4196 were
subjected to energy minimization and submitted to the MIB2 server for evaluation of the ion binding score (IBS) of individual amino acid residues and the number of ion binding poses (NIBP) for Ca and Mg ions."

Please elaborate on the "ion binding score" and "number of ion binding poses" concepts and provide reference for the MIB2 server.

*Thank you for the proposal. We added the reference for the server* ***(Lu et al., 2022)*** *(line 228) and added the information: “****IBS values of individual residues are determined using sequence and structure conservation comparison with 409 and 209 respective templates from the PDB database for Ca^2+^ and Mg^2+^ (Lin et al., 2016) and assessing the similarity of the configuration of the residue to its configurations in known structures of its complexes with the given metal (Lu et al., 2012). Ion binding sites are determined by locally aligning the query protein with the metal ion-binding templates and calculating its score as the RMSD-weighted scoring function Z. The site is accepted if it has a scoring function Z>1, and based on the local 3D structure alignment between the query protein and the metal ion-binding template, the metal ion in the template is transformed into the query protein structure (Lin et al., 2016). The larger the IBS value, the higher the tendency of the residue to bind the ion. The larger the NIBP value, the larger the number of such complexes with acceptable structure****" (lines 225-234).*

9/ Lines 460-466: Nine structural models of RyR were selected, and then these are referred to in the text only with the pdb code. The reviewer understands that it would be difficult to recapitulate all conditions but either a table in the main manuscript file or a minimal description in the text following the pdb code would increase clarity and help readers to follow the content.

*Thank you for the proposal. We added a new Table 2 “****Model structures used for identifying the allosteric pathways*”** *following line 450 that contains the required information, and inserted a reference to it in the text at line 446* ***“****According to these criteria we selected five RyR1 model structures (****Table 2)…”***

10/ Line 467: "In the selected structures, we identified residues with high allosteric coupling intensities (ACI) for both the inhibition and activation network and compared them with residues important for ligand binding and gating of RyR (Table 2)."

Please define further the concept of "allosteric coupling intensities". The corresponding methods section appears to focus on the outputs of the OHM server without delving too much on the algorithm or principles followed. Is the allosteric coupling between neighboring residues, or reflect movement of the residues due to ligand binding? Is there a "reference" state or are the comparisons carried out within each allosteric state? This would help to introduce better the sections "The inhibition network" and "The activation network".

*Thank you for this suggestion. We have lately realized, considering both the server output and the original work of Wang et al. (2020), that a better term for the variable depicting the role of the residue in the allosteric pathway would be the residue importance RI rather than the ACI. The allosteric pathway is determined on the basis of the network of contacts between pairs of residues in the given structure. The more contacts are present between two residues, the higher is the probability that a perturbation will be propagated from one to the other residue (Eq. 3 of Wang et al. (2020)). An allosteric pathway is then defined as the pathway that transmits the signal the whole way from the allosteric site to the active site.*

*Based on this we have changed in the manuscript the term “allosteric coupling intensity” to “residue importance” throughout the text and figures of the manuscript. It should be underlined, that this change has no effect whatsoever on presented data and conclusions. We inserted the following formulation in the Results section:*

*“The term* ***residue importance*** *defines the extent to which the given residue is involved in the propagation of a perturbation from the allosteric site to the active site, i.e., the fraction of simulated perturbations transmitted through this particular residue.* ***The more contacts are present between two residues, the higher is the probability that a perturbation will be propagated from one to the other residue (Wang et al., 2020).****” (lines 439-443).*

*We also inserted the following formulations into the Methods section: “The simulation of the perturbation propagation was performed 10 000 times per structure and pathway to estimate the values of residue importance.” (lines 1090-1092), and* *we expanded the relevant sentence: “Allosteric pathways were traced using the server OHM (https://dokhlab.med.psu.edu/ohm/#/home, (Wang et al., 2020))****, in which the allosteric pathway is determined on the basis of the network of contacts between pairs of residues in the given structure.”*** *(lines 1079-1081).*

11/ Figure 8: The figure would be more meaningful if the pathways were drawn in the context of the 3D structure.

*Thank you for the proposal. The pathways described in Fig. 8 are too complex for description in the RyR 3D structure, therefore they were not presented in the original manuscript. However, to follow the reviewer’s proposal we have illustrated the pathways observed in the inactivated RyR1 channel (7tdg) and the open RyR2 channel (7u9) in S1 Fig and added the corresponding movies S1 and S2 Movie. These RyR structures were selected for displaying both the intra- and inter-monomeric inactivation pathways.*

12/ Lines 610-612: "The structure of the inactivated RyR2 has not been determined yet; however, it is plausible to suppose that it exists at high concentrations of divalent ions and differs from the inactivated RyR1 structure by the extent of EF-hand - S23* coupling. "

The speculation would be more fit for the discussion section.

*Thank you for the proposal; however, the sentence introduces a logical supposition, necessary there for reasoning on the construction of the model. We reformulated the sentence to: “****In the absence of a structure of the inactivated RyR2, the model assumes that such a structure exists at high concentrations of divalent ions and differs from the inactivated RyR1 structure by the extent of EF-hand - S23* coupling.****” (lines 570-572).*

13 Lines 617-619: Closed and primed macrostates could be combined into a single closed macrostate of the model since both are closed and cannot be functionally distinguished at a constant ATP concentration.

The rationale for combining closed with primed does not seem a good idea, especially since the authors also mention that "the primed state is structurally very close to the open state" (lines
925-926). If the COI model is based on the structural findings, in principle it seems that primed should be treated separately.

*Thank you for the proposal. The use of both the closed and primed states was crucial for solving the model. As a matter of fact, although the primed and closed states are in part structurally different, functionally they are identical, that is, closed. Consequently, to be distinguished in a functional model we would need to incorporate single-channel data obtained under conditions when the ratio of closed and primed channels was modulated under otherwise identical conditions. Unfortunately, such a set of data, for instance at a varying ATP concentration for a range of cytosolic Ca^2+^ concentrations, does not exist for either RyR1 or RyR2 channels. Moreover, while there are several RyR1 high-resolution structures in the primed state (such as the 7tzc that we used; 2.45 Å; Melville et al. (2022)), the resolution of the corresponding RyR2 structures (6jg3, 6jh6, 6jhn; 4.5 – 6.1 Å; Chi et al. (2019)) is not sufficient for determination of allosteric pathways**. Fortunately, however, the two sets of conditions for RyR2 open probability data that were available in the literature turned out to represent activation of channels either selectively from the closed state (Fig. 10C), or almost selectively from the primed state (Fig. 10A, B). This allowed us to interpret the difference in the allosteric coefficients as a consequence of this fact.*

*To better clarify the idea, the corresponding text of the Discussion was modified as follows (lines 924-929): “RyR* ***channels can be considered mostly*** *in the primed state* ***under these conditions*** *since the binding of ATP analogs induces the primed structural macrostate in RyRs* ***even in the absence of Ca^2+^*** *(Cholak et al., 2023).* ***Fortunately, the two sets of conditions for RyR2 open probability data that were available in the literature turned out to represent activation of channels either selectively from the closed state (Fig. 10C), or selectively from the primed state (Fig. 10A, B)****.”, and “construction of such a model is at present hampered by the lack of open probability data at a sufficiently wide range of experimental conditions* ***and the absence of high-resolution structures of WT RyR2 in the primed state”*** *(lines 932-935)****.***

14/ Line 619. Please define the "COI" acronym. I assume it is closed, open and inactivated but this is not mentioned.

*We thank the reviewer for noticing the insufficiency. We expanded the specific sentence as follows: Therefore, we constructed the model of RyR operation, termed the COI* ***(closed-open-inactivated)*** *model, in which we assigned a functional macrostate corresponding to each of the closed, open, and inactivated structural macrostates (Figure 9A)” (line 579).*

15/ Figure 9: The diagrams are difficult to follow. Something that could improve it is to differentiate more between open and closed gates, but further elaboration would help the reader.

*We thank the reviewer for paying attention to details. The open state was differentiated in Figure 9 (after line 600) by adding a pore opening to the gate.*

*To elaborate on the gating transitions and to keep the manuscript concise, we added a new S2 Fig, which illustrates the relationship between the ion binding within macrostates and the transitions between macrostates.*

*Nevertheless, for the complexity of the model, which would need a multidimensional presentation, we had to limit the illustration to only the binding of the first ions at the binding sites. We hope that it will help the reader to grasp the principle of the model function more easily.*

16/ One comment is that the manuscript is too long; the manuscript exceeds the typical length required by most journals. To enhance its suitability for publication, the content needs to be synthesized and streamlined. The manuscript is written for an audience specialized in the RyR field and may be challenging for outsiders or for readers unfamiliar with structure and/or biophysical models.

*We thank the reviewer for opening this problem. The specific contribution to the understanding of RyR operation communicated by this manuscript was achieved by the synergy of approaches coming from different fields of RyR research – the structural, the functional, and the synthetic/systems ones. This needed deep immersion into complex studies performed over several decades to unwrap their complementary contributions. Only then we could synthesize the stepwise advances and integrate the mosaic of partial discoveries into the COI model. When conceptualizing the manuscript we were also considering a two-paper version, one on structural aspects and the other on modeling aspects. We realized that the two papers would need to have a very high overlap at the allosteric mechanism to be understandable in separation and would be difficult to publish in the same journal. We also anticipated a typical side effect that structuralists and modelers would read just their parts and would not appreciate enough the feedback from alternative views – how to design and interpret future structural, functional, and modeling studies.*

*Compacting the manuscript would be extremely difficult for us. In our view, the dense text would make it even more challenging for readers unfamiliar with some of the numerous approaches used here, as often happens to prominent multidisciplinary journals. Maybe it would be possible with the help of AI, but for now, we prefer to remain authentic.*

*Nevertheless, we made some effort. To shorten the manuscript, we have removed the paragraph describing the timeline of the search for the RyR inhibition site that was originally on lines 126-151 and replaced it with the paragraph on lines 130-135: “****The regulatory domains involved in both, activation and inactivation of RyRs (Figure 1) are located in the C-terminal quarter of the RyR. The Central domain participates in the Ca^2+^ binding activation site; the C-terminal domain bears several residues of Ca-, ATP- and caffeine-binding activation sites; the U-motif participates at the ATP- and caffeine-binding sites; the EF-hand region contains the putative Ca-binding pair EF1 and EF2; and the S23 loop bears one residue of the caffeine-binding site and two residues interacting with the EF-hand region of a neighboring monomer (Samso, 2017; Hadiatullah et al., 2022)****”. We also removed the statements about the proposed kinetic mechanism of inactivation by Nayak et al. (2022), originally on lines 175-184. Finally, we removed the discussion of the work of Gomez et al. (2016) originally on lines 882-889, since it fully overlapped with the statements in Results on lines 358-367 (now lines 338-347). We also moved the text of the subsection “Relationship between the COI model and RyR allosteric pathways” (originally lines 670-685) into subsection “Construction of the model of RyR operation”, lines 591-595 and 639-663 of the revised version.*

17/ Another comment is the limited consideration of two relevant published works. One is by Chirasani et al. (2024), focused on allosteric pathways similar to the ones described here. The other work is by Nayak et al (2024), with cryo-EM structures of RyR1 focused on the interplay with Mg2+ and Ca2+. Overall, the manuscript would be strengthened by incorporating such related results in the literature.

*We thank the reviewer for the concerns, but we cannot fully agree. The paper of Chirasani et al. (2024 ) was cited in the manuscript as its online-first version, Chirasani et al. (2023). The manuscript now refers to the printed version proposed by the reviewer. The Chirasani et al. work was discussed on lines 870-881. The paper concentrates on the interaction between the EF-hand region and the S23 segment and its effect on RyR inactivation, which we referenced in the manuscript, but not on the allosteric pathways as mentioned by the reviewer. To broaden the consideration of this important work, we have introduced a more detailed discussion of Chirasani et al. (2024 ) by adding the following text to the manuscript: Lines 878-886: “****Based on their structural analysis of the open RyR1 structure 5tal,*** *Chirasani et al. (2024 )* ***proposed that narrowing the gap between the EF-hand domain and S23 loop, resulting in H-bonding interactions between the EF-hand residue K4101 and the S23 loop residue D4730, and those between the EF-hand residues E4075, Q4076, D4079 and the S23 loop residue R4736, is a consequence of the binding of Ca^2+^ to the EF-hands. However, our*** *PDBePISA analysis revealed* ***a*** ***similar number of interactions*** *between the EF-hand region and the S23* loop* ***not only in open and inactivated but also in primed*** *RyR1 structures (Figure 3).* ***The presence of EF hand-S23* hydrogen bonds in the primed and open RyR1 structures suggests that the proximity of the EF-hand domain and S23* loop is a structural trait distinguishing RyR1 from RyR2, not a consequence of Ca^2+^ binding to the EF hand.****”*

*The data and ideas of the illuminating work of Nayak et al. (2024) were discussed and referred to in the manuscript in several places, originally lines 74, 77, 164 (Introduction), 311, 340 (Results), 892-893, and 971 (Discussion). To broaden consideration of this work, we have expanded the discussion of this paper by adding the text shown in bold into the Introduction: “Recent studies reporting RyR structure at a* ***high divalent ion concentration provide only indirect support for the molecular mechanism of Ca^2+^/Mg^2+^-dependent inactivation. Wei et al. (2016) and Nayak et al. (2024) observed a change in the conformation of the RyR1 EF-hands in the presence of 100 µM Ca^2+^ and 10 mM Mg^2+^, respectively, compared to low-calcium or low-magnesium conditions*.***” (lines 136-139) and in the Discussion (lines 887-889): “The recent RyR1 structure 7umz* ***(Nayak et al., 2024) provided evidence of Mg^2+^ ion bound in the RyR activation site, thus confirming the functional studies that established competition between Ca^2+^ and Mg^2+^ at this activation site (Laver et al., 1997; Zahradnikova et al., 2003; Zahradnikova et al., 2010).****”*

Reviewer 3:

Minor comment: While I am not an expert in allosteric model construction and therefore cannot fully assess their methodological approach, I observed that the authors fixed a number of parameters to achieve model convergence. A more detailed explanation of the rationale behind these fixed parameters would enhance clarity. Currently, these parameters are not clearly specified in the text and are somewhat obscured by the broader description of all parameters included in the model.

*We thank the reviewer very much for this comment, which made us realize that the relevant sections were written in a too technical manner, without sufficient explanation of the ideas behind the derivation and optimization of the model. To clarify the rationale of this process, we have rewritten the subsection “Derivation of the model open probability equation” and the section “Description of RyR operation by the COI model”. In the subsection “Derivation of the model open probability equation”, we have explained the simplification of the full set of equations (Eqs. 3A-C) into Eqs. 4A-C (lines 639 – 663). In the section “Description of RyR operation by the COI model”, we have explained the extent of over-parametrization and the rationale of reducing it by three methods: combining the data into groups with common parameter values; eliminating parameter interdependence by fixation of one parameter at a preset value taken from the literature or postulated a priori; and sharing parameter values between data groups when no significant difference between these values was observed (lines 678-682, 699-707, 716-737).*

*We hope that these changes make the manuscript more comprehensible.*

REFERENCES

Chi, X., D. Gong, K. Ren, G. Zhou, G. Huang, J. Lei, Q. Zhou, and N. Yan. 2019. Molecular basis for allosteric regulation of the type 2 ryanodine receptor channel gating by key modulators. *Proceedings of the National Academy of Sciences of the United States of America*. 116:25575-25582.

Chirasani, V.R., M. Elferdink, M. Kral, J.S. Carter, S. Heitmann, G. Meissner, and N. Yamaguchi. 2024 Structural and functional interactions between the EF hand domain and S2-S3 loop in the type-1 ryanodine receptor ion channel. *The Journal of biological chemistry*. 300:105606.

Cholak, S., J.W. Saville, X. Zhu, A.M. Berezuk, K.S. Tuttle, O. Haji-Ghassemi, F.J. Alvarado, F. Van Petegem, and S. Subramaniam. 2023. Allosteric modulation of ryanodine receptor RyR1 by nucleotide derivatives. *Structure*. 31:790-800 e794.

Dashti, A., G. Mashayekhi, M. Shekhar, D. Ben Hail, S. Salah, P. Schwander, A. des Georges, A. Singharoy, J. Frank, and A. Ourmazd. 2020. Retrieving functional pathways of biomolecules from single-particle snapshots. *Nature communications*. 11:4734.

Gomez, A.C., T.W. Holford, and N. Yamaguchi. 2016. Malignant hyperthermia-associated mutations in the S2-S3 cytoplasmic loop of type 1 ryanodine receptor calcium channel impair calcium-dependent inactivation. *American journal of physiology*. 311:C749-C757.

Hadiatullah, H., Z. He, and Z. Yuchi. 2022. Structural Insight Into Ryanodine Receptor Channelopathies. *Frontiers in pharmacology*. 13:897494.

Laver, D.R., T.M. Baynes, and A.F. Dulhunty. 1997. Magnesium inhibition of ryanodine-receptor calcium channels: Evidence for two independent mechanisms. *J.Membrane.Biol*. 156:213-229.

Lin, Y.F., C.W. Cheng, C.S. Shih, J.K. Hwang, C.S. Yu, and C.H. Lu. 2016. MIB: Metal Ion-Binding Site Prediction and Docking Server. *Journal of chemical information and modeling*. 56:2287-2291.

Lu, C.H., C.C. Chen, C.S. Yu, Y.Y. Liu, J.J. Liu, S.T. Wei, and Y.F. Lin. 2022. MIB2: metal ion-binding site prediction and modeling server. *Bioinformatics*. 38:4428-4429.

Lu, C.H., Y.F. Lin, J.J. Lin, and C.S. Yu. 2012. Prediction of metal ion-binding sites in proteins using the fragment transformation method. *PLoS One*. 7:e39252.

Mackrill, J.J. 2022. Evolution of the cardiac dyad. *Philosophical transactions of the Royal Society of London. Series B, Biological sciences*. 377:20210329.

Melville, Z., K. Kim, O.B. Clarke, and A.R. Marks. 2022. High-resolution structure of the membrane-embedded skeletal muscle ryanodine receptor. *Structure*. 30:172-180 e173.

Nayak, A.R., W. Rangubpit, A.H. Will, Y. Hu, P. Castro-Hartmann, J.J. Lobo, K. Dryden, G.D. Lamb, P. Sompornpisut, and M. Samso. 2024. Interplay between Mg(2+) and Ca(2+) at multiple sites of the ryanodine receptor. *Nature communications*. 15:4115.

Nayak, A.R., and M. Samso. 2022. Ca(2+) inactivation of the mammalian ryanodine receptor type 1 in a lipidic environment revealed by cryo-EM. *eLife*. 11.

Rossi, D., A.M. Scarcella, E. Liguori, S. Lorenzini, E. Pierantozzi, C. Kutchukian, V. Jacquemond, M. Messa, P. De Camilli, and V. Sorrentino. 2019. Molecular determinants of homo- and heteromeric interactions of Junctophilin-1 at triads in adult skeletal muscle fibers. *Proceedings of the National Academy of Sciences of the United States of America*. 116:15716-15724.

Samso, M. 2017. A guide to the 3D structure of the ryanodine receptor type 1 by cryoEM. *Protein science : a publication of the Protein Society*. 26:52-68.

Wang, J., A. Jain, L.R. McDonald, C. Gambogi, A.L. Lee, and N.V. Dokholyan. 2020. Mapping allosteric communications within individual proteins. *Nature communications*. 11:3862.

Wei, R., X. Wang, Y. Zhang, S. Mukherjee, L. Zhang, Q. Chen, X. Huang, S. Jing, C. Liu, S. Li, G. Wang, Y. Xu, S. Zhu, A.J. Williams, F. Sun, and C.C. Yin. 2016. Structural insights into Ca(2+)-activated long-range allosteric channel gating of RyR1. *Cell research*. 26:977-994.

Zahradnikova, A., M. Dura, I. Gyorke, A.L. Escobar, I. Zahradnik, and S. Gyorke. 2003. Regulation of dynamic behavior of cardiac ryanodine receptor by Mg2+ under simulated physiological conditions. *American journal of physiology*. 285:C1059-1070.

Zahradnikova, A., I. Valent, and I. Zahradnik. 2010. Frequency and release flux of calcium sparks in rat cardiac myocytes: a relation to RYR gating. *The Journal of general physiology*. 136:101-116.

Zheng, W., and H. Wen. 2020. Investigating dual Ca(2+) modulation of the ryanodine receptor 1 by molecular dynamics simulation. *Proteins*. 88:1528-1539.
