# Supplementary material for: Structure-based mechanism of RyR channel operation by calcium and magnesium ions
Source: PLoS Comput Biol. 2025 Apr 29;21(4):e1012950. doi: 10.1371/journal.pcbi.1012950 (PMC12119028; doi:10.1371/journal.pcbi.1012950)
Supplement: Attachment — Submitted filename: Comments_HBAGDec2024.docx [file pcbi.1012950.s014.docx]

The manuscript has been greatly improved compared to the previous submission and the authors responded appropriately to all comments raised.

One issue remaining pertains to the accuracy of the model in key regions for the analysis and in the reporting of the quality of these models, as models' quality are critical for allosteric pathway calculations, as detailed in points 6,7,and 8 below.

I recommend publication with these issues addressed.

Specific comments:

1. **Lines 106-108**: *Recently, mathematical modeling of the cardiac calcium release site (Iaparov et al., 2022****) confirmed*** *that Mg2+ ions could at the same time act as the negative competitor at the calcium activation site and as an inhibitor at the inhibition site.*

Please mention the **experiments** in conjunction with mathematical modeling that confirmed the above-mentioned effects of Mg2+. If the inference is only drawn from mathematical modeling, I suggest authors replace **confirmed** with **showed/demonstrated.**

2. **Lines 190-191**: *All structures and open probabilities analyzed here are listed in* ***Extended View Table S1****.*

Is the extended view table S1 the same as S1 file? I do not seem to find Table S1 with open probabilities of the structures listed.

3. **Lines 137-138:** *Wei et al. (2016) and* ***Nayak et al. (2004)*** *observed a change in the*

Nayak et al. (2004) is not on the reference list. Could it be 2024 instead of 2004?

4. **Lines 225-228***: IBS values of individual residues are determined using sequence and structure conservation comparison with* ***409 and 209 respective templates from the PDB database for Ca2+ and Mg2+*** *(Lin et al., 2016) and assessing the similarity of the configuration of the residue to its configurations in known structures of its complexes with the given metal (Lu et al., 2012).*

AlphaFill databank (<https://alphafill.eu/>) contains all AlphaFold models with ligands, cofactors and **metal ions** built into them using AlphaFill algorithm. Given the recent successes of AlphaFold2 in accurate protein structure prediction and related methods based on it, the 409 and 209 templates from the PDB database could potentially be extended to include templates from AlphaFill database for Ca2+ and Mg2+ for calculation of IBS and NIBP values.

5. Note on the use of abbreviations:

For readability to general audience, I suggest authors expand the abbreviations when they first use them and not leave the task to the readers.

e.g. Lines 241-242: **RyR1scr and RyRscr**

scr stands for scrambled or something else should be mentioned in the text.

**Lines 415, 418**: RyR1-O, RyR2-O, RyR1-I

The codes O for open and I for inactivated were introduced very early in the text and it becomes difficult to immediately interpret RyR1-O as open state after reading through various codes and abbreviations mentioned earlier in the text.

6. **Line 413 :** Interaction of EF-hand region with S23 loop and **Fig. 5**

Most of the residues analyzed in this section and Fig. 5 have poor fit to the cryo-EM map for PDBs 7m6l and 7ua9 according to the validation report for these PDBs. This could be due to either missing density for that residue or the residue not properly fitted in the density map. In the PDB validation report, in the residue property plot a red diamond above a residue indicates a poor fit to the EM map for that residue (all-atom inclusion < 40%) also quantified by low Q-scores. All the residues analyzed here from 7m6l and many from 7ua9 have red diamond above them. A quick visualization of map and model in Coot at the analyzed region also shows poor fit (attached for 7m6l and 7ua9 at the interaction interface). **Therefore, I suggest authors to be careful in their analysis when interpretating side-chain interactions such as hydrogen bonds and salt-bridges between poorly fit and modeled residues in the density maps and their subsequent use in modeling the channel’s behavior.**

**I also suggest authors to find evidence from other RyR1 and RyR2 cryo-EM structures where these residues have interpretable density** to support the statements in this section such as “***This confirms the results of the distance analysis, namely that RyR1 structures can make hydrogen bonds between E4075 and R4736* and between K4101 and D4730*, while the corresponding RyR2 structures can make a hydrogen bond only between K4056 and E4659* but not between D4030 and R4665* (hRyR2 numbering)****.*”

7. **Lines 1025-1034: Editing and optimization of structures**

What programs were used to reconstruct unresolved loops and add missing atoms to the incomplete residues? It is not clearly mentioned in the text. Was it not possible to predict and build the missing regions, loops and residues in the structures using AlphaFold2 or recent v3 as in most cases it can predict structures at the side-chain level with experimental accuracy. It would be interesting to see the AlphaFold predictions for the unresolved DR1 loop modeled here as polyglycine loop.

8. Significant results of the manuscript are derived from calculations done on side-chain of residues including calculating IBS and NIBP values, calculation of allosteric paths and potentially distance calculations which therefore requires modeling of side-chains with proper stereochemistry. One of the weaknesses of the manuscript is that little to no evidence has been provided on the geometry or stereochemical accuracy (standard vs non-standard rotamers, Ramachandran plots, clash score and other metrics) of the residues in the structures/sub-structures that have been used in the above-mentioned calculations and subsequent analysis.

Most of the cryo-EM structures have missing regions/residues which are reconstructed/repaired and energy minimized by authors in this work. The addition of missing regions most often introduces steric clashes which when removed through energy minimization can introduce rotamer and Ramachandran outliers indicating non-standard geometries for those residues. Allosteric paths involving such residues may undermine the model of RyR operation presented in the manuscript.

For residues/regions/sub-structures involved in various/important allosteric paths given in Tables, for residues used for IBS and NIBP and potentially distance calculations, I suggest authors to provide Q-scores signifying their fit to density maps. Further, for these regions/residues/sub-structures repaired and energy minimized by the authors, I suggest providing MolProbity reports mentioning clash scores, rotamer and Ramachandran outliers. In case MolProbity reports recommends corrections, I suggest authors to run allosteric path calculations (OHM server) on corrected sub-structures (corrected for residue clashes, rotamer and Ramachandran outliers) and reinterpret the allosteric paths if the resultant paths differ from the one analyzed in the manuscript.
