# Supplementary material for: Structure-based mechanism of RyR channel operation by calcium and magnesium ions
Source: PLoS Comput Biol. 2025 Apr 29;21(4):e1012950. doi: 10.1371/journal.pcbi.1012950 (PMC12119028; doi:10.1371/journal.pcbi.1012950)
Supplement: Attachment — Submitted filename: Response_PCOMPBIOL-D-24-02096_PLoS.docx [file pcbi.1012950.s016.docx]

**Responses to reviewers.**

We thank the reviewers for their efforts to make the manuscript better. We have modified the manuscript in line with the comments of the reviewers and removed several typographic errors and minor omissions.

**Reviewer #1:** This is a technical paper on an important topic for the RyR channel and ion channel communities, but it is of more limited interest to the general public as no direct applications can be gleaned from it.

Although the article is based on various technical approaches, its significance is in the original combination of structural and functional data that allowed us to break the barrier between structuralists and functionalists. This approach might be of general interest, although the most direct application is in the field of multimeric membrane proteins such as ion channels and receptors.

We have modified the corresponding sentence (ll. 967-969): “This approach may find use in deciphering the allosteric mechanisms of **multimeric proteins such as** ion channels **or receptors** in general.”

The manuscript has been greatly improved compared to the previous submission and the authors responded appropriately to all comments raised.

One issue remaining pertains to the accuracy of the model in key regions for the analysis and in the reporting of the quality of these models, as models' quality are critical for allosteric pathway calculations, as detailed in points 6,7, and 8 below.

I recommend publication with these issues addressed.

We thank the reviewer for the positive evaluation and the constructive comments.

**Specific comments:**

1. **Lines 106-108**: Recently, mathematical modeling of the cardiac calcium release site (Iaparov et al., 2022**) confirmed** that Mg2+ ions could at the same time act as the negative competitor at the calcium activation site and as an inhibitor at the inhibition site.

Please mention the **experiments** in conjunction with mathematical modeling that confirmed the above-mentioned effects of Mg2+. If the inference is only drawn from mathematical modeling, I suggest authors replace **confirmed** with **showed/demonstrated.**

According to the reviewer’s suggestion, we changed “confirmed” to “**demonstrated**” (line 105).

2. **Lines 190-191**: All structures and open probabilities analyzed here are listed in **Extended View Table S1**.

Is the extended view table S1 the same as S1 file? I do not seem to find Table S1 with open probabilities of the structures listed.

Thank you for noticing the error, which was due to the different formats between the two journals. We corrected “Extended View Table S1” to “**S1 File**” (line 183).

3. **Lines 137-138:** Wei et al. (2016) and **Nayak et al. (2004)** observed a change in the

Nayak et al. (2004) is not on the reference list. Could it be 2024 instead of 2004?

Thank you for noticing the error. The text now cites the correct reference (line 133).

4. **Lines 225-228**: IBS values of individual residues are determined using sequence and structure conservation comparison with **409 and 209 respective templates from the PDB database for Ca2+ and Mg2+** (Lin et al., 2016) and assessing the similarity of the configuration of the residue to its configurations in known structures of its complexes with the given metal (Lu et al., 2012).

AlphaFill databank (<https://alphafill.eu/>) contains all AlphaFold models with ligands, cofactors and **metal ions** built into them using AlphaFill algorithm. Given the recent successes of AlphaFold2 in accurate protein structure prediction and related methods based on it, the 409 and 209 templates from the PDB database could potentially be extended to include templates from AlphaFill database for Ca2+ and Mg2+ for calculation of IBS and NIBP values.

We thank the reviewer and agree that using the AlphaFill databank would greatly improve the precision of the MIB2 server. However, the templates used in this server were fixed so that they cannot be changed. We have changed the wording to clarify this point (shown in bold; ll. 215-221):

“**The server determines the** IBS values of individual residues using sequence and structure conservation comparison with 409 and 209 **pre-set** templates from the PDB database for Ca^2+^ and Mg^2+^**, respectively** [52], and assessing the similarity of the configuration of the residue to its configurations in known structures of its complexes with the given metal [53]. **The server determines potential ion-binding** sites by locally aligning the query protein with the metal ion-binding templates and calculating its score as the RMSD-weighted scoring function Z.”

5. Note on the use of abbreviations:

For readability to general audience, I suggest authors expand the abbreviations when they first use them and not leave the task to the readers.

e.g. Lines 241-242: **RyR1scr and RyRscr**

scr stands for scrambled or something else should be mentioned in the text.

We thank the reviewer for noticing the error and have corrected the typo and added the explanations (l. 231-234): “ANOVA analysis indicated that IBS of EF1 for Ca^2+^ ions was significantly higher in RyR1 and RyR2 **structures** than in the **scrambled RyR1 structure** RyR1scr (p = 0.0128 and p < 0.001) but for Mg^2+^ ions only the difference between RyR2 and **RyR1scr** was significant (p < 0.001). EF1 and EF2 bind Ca^2+^ equally in both isoforms.”;

**Lines 415, 418**: RyR1-O, RyR2-O, RyR1-I

The codes O for open and I for inactivated were introduced very early in the text and it becomes difficult to immediately interpret RyR1-O as open state after reading through various codes and abbreviations mentioned earlier in the text.

We have gratefully changed the text as follows (l. 401-406): “To better understand the above-disclosed difference in the displacement of the EF-hand region and S23 loop between RyR1 and RyR2 structures (Fig 4 D and E), we aligned the **open** RyR1 and RyR2 model structures on their more static S23* loop (Fig 5). We compared here only the open states, since according to the distance analysis (Fig 2) the distances in the closed states are too large, the inactivated RyR2 structure is not available, and the distances D1 and D2 are similar **in the open and inactivated** RyR1.“ We have also added “**open**” into the description of the structures in the legend to Fig 5.

6. **Line 413 :** Interaction of EF-hand region with S23 loop and **Fig. 5**

Most of the residues analyzed in this section and Fig. 5 have poor fit to the cryo-EM map for PDBs 7m6l and 7ua9 according to the validation report for these PDBs. This could be due to either missing density for that residue or the residue not properly fitted in the density map. In the PDB validation report, in the residue property plot a red diamond above a residue indicates a poor fit to the EM map for that residue (all-atom inclusion < 40%) also quantified by low Q-scores. All the residues analyzed here from 7m6l and many from 7ua9 have red diamond above them. A quick visualization of map and model in Coot at the analyzed region also shows poor fit (attached for 7m6l and 7ua9 at the interaction interface). **Therefore, I suggest authors to be careful in their analysis when interpretating side-chain interactions such as hydrogen bonds and salt-bridges between poorly fit and modeled residues in the density maps and their subsequent use in modeling the channel’s behavior.**

We agree with the reviewer that the sidechains of important residues are either not resolved or do not fit well into the electron density map. Therefore we made changes to address this point: We added a new Supplemental figure (Figure 1; S1 Fig in the manuscript), illustrating the overall differences between RyR1 and RyR2 of all investigated structures in the primed, open, and inactivated states, changed the title of Fig. 5 in the manuscript to “**Possible** interactions”, the description of panel B to “**Possible** residue interactions” and added the text “**The spatial arrangement of all primed, open, and inactivated structures is compared for RyR1 and RyR2 in S1 Fig.**” to the legend. We also changed the main text to indicate that we talk about inferences from the positions of the CA atoms and not of sidechains (ll. 408-412): “In RyR2, **the backbones of** both EF1 and EF2 are diverted downward relative to their counterparts in RyR1. **Consequently**, the D4030 of RyR2 (the equivalent of E4075 in RyR1) withdraws from **interacting** with S23* (Fig 5 B, C). **A similar change in the orientation of the EF-hand relative to the S23 loop was observed in all primed and open RyR2 structures examined (see S1 Fig for the** **visual comparison of all evaluated primed, open, and inactivated RyR1 and RyR2 structures).** “ We have also stressed the fact that CA atoms were used in the sentence (ll. 269-271): “Nayak and Samsó [22] measured the distance between the EF-hand region and the S23* loop as the distance between **the Cα atoms of** pairs of residues allowing direct interaction at sufficient proximity.”

Figure 1 (S1 Fig). A detailed view of the EF-hand region and the S23 loop taken from the open, primed and inactivated RyR structures aligned at their S23 loops. The different orientation of the EF-hand relative to the S23 loop in RyR1 *vs.* RyR2 is indicated by the red double arrows. Note the similar orientation of the EF-hand region relative to the S23 loop in all structures of the same isoform.

S1 Fig complements Figure 5.

I also suggest authors to find evidence from other RyR1 and RyR2 cryo-EM structures where these residues have interpretable density to support the statements in this section such as “*This confirms the results of the distance analysis, namely that RyR1 structures can make hydrogen bonds between E4075 and R4736* and between K4101 and D4730*, while the corresponding RyR2 structures can make a hydrogen bond only between K4056 and E4659* but not between D4030 and R4665* (hRyR2 numbering).*”

Unfortunately, most of the published structures do not have all atoms attributed. Considering the contiguous portions of the electron densities shown in Figure 2 below, it is apparent that the relative position of the EF-hand region and the S23 loop in the RyR2 channel does not allow electrostatic interactions of S23 with the sequence preceding the EF1 loop but it may allow such interactions between S23 and the sequence following EF1 loop.

Figure 2. The structures 7m6l (left) and 7ua9 (right) are superimposed with their corresponding electron density maps EMD-23699 and EMD-26416 at a lower threshold (Chimera levels 0.045 and 0.085, respectively). The EF-hand loops and S23 loop are indicated. The red ovals indicate the regions, in which the electron densities of the chain A and D overlap.

Thus we have reformulated the text in the manuscript (ll. 412-417):

“This confirms the results of the distance analysis, namely that **the formation of** hydrogen bonds between **K4101 and D4730*** and between **E4075 and R4736*** **is possible in primed, open, and inactivated RyR1 structures**, while **the formation of** a hydrogen bond **is possible** only between K4056 and E4659* **(hRyR2 equivalents of K4101 and D4730*)** but not between D4030 and R4665* (hRyR2 **equivalents of E4075 and R4736*) in the corresponding primed and open RyR2 structures**.” We also spelled out the state names on ll. 294-296: “These results indicate that the glutamate-arginine* (E4075 and R4736*) distance D1 between the EF-hand and S23* regions is too large in all RyR2 structures, in contrast to the RyR1 **primed, open, and inactivated** states, in which the residues may potentially interact and transmit the binding signal.”

7. **Lines 1025-1034: Editing and optimization of structures**

What programs were used to reconstruct unresolved loops and add missing atoms to the incomplete residues? It is not clearly mentioned in the text.

As originally stated, unresolved loops were first reconstructed in Chimera by using MODELER and optimized using MODELER and/or MODLOOP. In the revised version we have clarified the procedure (ll. 1011-1016): “In model structures used for the determination of allosteric pathways, the short unresolved loops were reconstructed to correspond to the known sequence, and the missing long part of the unresolved DR1 region was replaced by a polyglycine loop, **using the MODELLER [****86****] add-on to Chimera**. The reconstructed loops were optimized using MODELLER [86] or the server MODLOOP (<https://modbase.compbio.ucsf.edu/modloop/> [87, 88]). The edited structures were repaired by **replacing** incomplete residues **using the Dunbrack library [****89****].**”

Was it not possible to predict and build the missing regions, loops and residues in the structures using AlphaFold2 or recent v3 as in most cases it can predict structures at the side-chain level with experimental accuracy. It would be interesting to see the AlphaFold predictions for the unresolved DR1 loop modeled here as polyglycine loop.

Unfortunately, Alphafold3 is not able to correctly predict the DR1 region, as shown in Figure 3 below for the models of hRyR1 (A) and hRyR2 (B) cores with the best score. The DR1 region is shown in red. Individual instances of the model differed in the number and sequence positions of helices. The RyR2 DR1 loops contained additionally one β hairpin. The main problems were in the extremely variable positioning of the secondary structure elements relative to the remaining structure in individual instances of the Alphafold3 models, and in the fact that the large unstructured loops almost always crossed the membrane.

Figure 3. AlphaFold3 predictions of the RyR core for sequences of human RyR1 (A) and RyR2 (B). For clarity, one monomer is shown in isolation from the remaining protein (left). The DR1 region is shown in red.

If the loops were modeled separately, the predicted models consisted solely of a random coil without the helix segments. We have a similar experience with predictions of other such long loops in ion channels.

We have also made AlphaFold3 predictions of the RyR2 core in which the DR1 region was replaced by 11 glycine residues. Figure 4 below shows the comparison of domain positions relative to the original experimental structures 7ua9 (open) and 7u9q (closed) for two models used in the manuscript (open 7ua9 and closed 7ua5) and for 10 instances of the AlphaFold3 hRyR2 model. The AlphaFold3 predictions were more similar to the closed than to the open channel conformation but all models created by AlphaFold had systematic deviations from the experimental structures as well as from the models used in the manuscript, especially in the positions of the S23 loop, S45 segment, and S6 helix relative to the central domain. Additionally, Alphafold3 models of both RyR1 and RyR2 cores had a ClashScore > 10 and a significant fraction of poor rotamers (1.7 – 2.5%), although they had only a low number of Ramachandran outliers.

Figure 4. Deviations of the position of RyR2 core domains of manually created RyR models (7ua9, 7ua5) and AlphaFold3 models (AlphaFold) relative to the central domain of a closed 7U9Q and open 7UA9 RyR2 channel structure.

8. Significant results of the manuscript are derived from calculations done on side-chain of residues including calculating IBS and NIBP values, calculation of allosteric paths and potentially distance calculations which therefore requires modeling of side-chains with proper stereochemistry. One of the weaknesses of the manuscript is that little to no evidence has been provided on the geometry or stereochemical accuracy (standard vs non-standard rotamers, Ramachandran plots, clash score and other metrics) of the residues in the structures/sub-structures that have been used in the above-mentioned calculations and subsequent analysis.

Thank you for suggesting MolProbity for the analysis and repair of structures.

Regarding the calculations of the IBS and NIBP values, it has to be stressed that the original structures had a very low resolution and a significant number of missing atoms in the examined EF-hand loops, probably due to their high mobility. MolProbity analysis of the region used as input to the MIB2 server (corresponding to rRyR1 residues 4063 – 4196) showed that the optimized structures have a higher number of poor rotamers and Ramachandran outliers but a much-decreased number of clashes (see Figure 5 below). As a result, the MolProbity scores for the original and optimized structures were not significantly different and were even nominally lower in the optimized structures. Originally, we have also analyzed divalent ion binding in the original structures (which showed similar differences between RyR1 and RyR2, EF1 and EF2, and Ca^2+^ and Mg^2+^ binding as the optimized ones), but we preferred using the optimized structures to be able to compare the WT structures with the scrambled RyR1 structure.

Figure 5. The MolProbity and Clash scores of the 20 WT model structures used for calculation of the IBS and NIBP at different phases of optimization. The scores were calculated for structures with atom coordinates identical to the experimental structure (Original), structures after adding all missing atoms (Completed), and structures after minimizing model energy (Optimized). Note the somewhat increased MolProbity score and ClashScore after completing the structures and its significant decrease after optimization. The significance of differences was calculated with repeated measures ANOVA.

It has to be stressed that all distance calculations were performed on the original structures. Because of the limited resolution, the calculations were made on the positions of CA atoms, i.e., were independent of the rotamers involved.

Most of the cryo-EM structures have missing regions/residues which are reconstructed/repaired and energy minimized by authors in this work. The addition of missing regions most often introduces steric clashes which when removed through energy minimization can introduce rotamer and Ramachandran outliers indicating non-standard geometries for those residues. Allosteric paths involving such residues may undermine the model of RyR operation presented in the manuscript.

We acknowledge the limitations caused by optimizing the structures, as mentioned above by the reviewer. However, it has to be stressed that the steric clashes did not involve mainly the added missing regions but rather the original atoms and added missing atoms in the resolved residues. The MolProbity score in optimized structures was not significantly different either from the original structures or structures in which the partially resolved residues were completed but the missing regions were not reconstructed, while the ClashScore was significantly lower in optimized structures than either in the original structures or structures in which the partially resolved residues were completed but the missing regions were not reconstructed (Figure 6).

Figure 6. The MolProbity and Clash scores of the 9 WT structures used for calculation of the allosteric pathways at different phases of optimization. The scores were calculated for structures with atom coordinates identical to the experimental structure (Original), structures after adding all missing atoms in partially resolved residues but lacking the missing regions (Completed), and structures with completed missing regions and minimized model energy (Optimized). The significance of differences was calculated with repeated measures ANOVA.

For residues/regions/sub-structures involved in various/important allosteric paths given in Tables, for residues used for IBS and NIBP and potentially distance calculations, I suggest authors to provide Q-scores signifying their fit to density maps.

We respectfully disagree with the reviewer, since especially for the residues used for IBS and NIBP analysis, it cannot be supposed that they would fit into the electron density maps. This is due to their poor resolution (their sidechains are often not resolved in the structures). We would like to point out that the previous simulation studies, whether involving allosteric pathways (Chirasani, 2022), hydrogen bond formation (Chirasani,2024), or divalent ion binding (Zheng, 2020), also included atoms that were not resolved in the experimental structures and therefore were not inside the electron density map. We suppose that structures with a more correct geometry (i.e., more similar to the canonical EF-hand structure) would score higher in MIB2 calculations since at present in most of the Ca^2+^ and Mg^2+^ complexes provided by the server, these ions were coordinated only by 2 or 3 residues from the EF-hand, much less than the optimal 6 residues.

Further, for these regions/residues/sub-structures repaired and energy minimized by the authors, I suggest providing MolProbity reports mentioning clash scores, rotamer and Ramachandran outliers. In case MolProbity reports recommends corrections, I suggest authors to run allosteric path calculations (OHM server) on corrected sub-structures (corrected for residue clashes, rotamer and Ramachandran outliers) and reinterpret the allosteric paths if the resultant paths differ from the one analyzed in the manuscript.

We have characterized the structures used for the determination of metal-binding scores and allosteric pathways in terms of their stereochemical accuracy and geometry and provide the results as the supplementary file “S3 File” (described in ll. 1199-1206). However, to the best of our knowledge, it is not possible to repair the structures without introducing personal bias since there are always multiple ways how to improve the rotamers, especially in regions that are not resolved in the electron density map (i.e., when the residue in question was present in many different conformations in the sample). Additional bias, although with a smaller effect on the allosteric pathways, may be introduced while repairing the Ramachandran outliers. Unfortunately, the server for automatic structure repair (<https://pdb-redo.eu/>) is suitable only for structures obtained using X-ray crystallography. Therefore we decided to report the quality of the models in the Supplemental data but not to repair and reanalyze the models. Nevertheless, for the purpose of this rebuttal, we have partially repaired the model corresponding to the 7tdg structure, which is the most essential for the conclusions of the paper, to check for the effect of model quality on the allosteric pathways. For the repair of the model, we used the ISOLDE plugin of ChimeraX. In six different instances of the repaired model, we analyzed the inhibitory allosteric pathways. All versions of the repaired model provided the same pathways leading from the EF-hand loops to the gate - the one going through the S23 loop (inter-monomeric, which we interpret as causing direct inactivation of RyRs), and the one touching the ATP binding site (intra-monomeric, which we interpret as causing inactivation by the interaction between the activating Ca^2+^ ion and the inhibitory divalent ion, integrated at the ATP binding site). The differences between pathways of individual model instances were in (1) the relative abundance of the inter-monomeric pathway (5 - 27%), (2) the identity of the ATP binding site residue on the intra-monomeric pathway (K4211, K4214, or both), and (3) the fraction of the intra-monomeric pathway going through the S45 segment (60 - 100%). These fractions did not depend on the quality of the structure, as shown in Figure 7 below for the fraction of the inter-monomeric pathway:

Figure 7. The relationships between the MolProbity score, ClashScore, and the fraction of inter-monomeric inhibitory pathway in six different instances of the repaired model 7tdg. The figure shows six models but some of them provided identical clash score/MolProbity score (×).

There is not a clear dependence between the overall model quality as reported by the MolProbity score and the fraction of inter-monomeric allosteric pathways. On the other hand, an increased clash score leads to a decreased incidence of the inter-monomeric pathway. However, it has to be stressed that the repaired structures still had a relatively large number of rotamer and Ramachandran outliers and it is questionable whether it is possible to model all atoms of the RyR core with idealized geometry while maintaining a sufficiently low RMSD of the CA atoms between the original reported structures and the models.

We reported the quality of the models in the supplementary S3 File, as stated in the Methods (ll. 1043-1045 and 1069-1070): “**The quality of models as determined by the MolProbity server [****92****] is reported in the S3 File.**” We acknowledged the above-mentioned limitations in the Discussion (ll. 924-932):

“**The determination of allosteric pathways was performed on models derived from cryo-EM structures with suboptimal resolution. Especially the regions of the EF-hand loops were not well resolved, and multiple residues of the structure were either represented by alanines or did not satisfactorily fit into the electron density map. Since the topology of the unresolved DR1 region is not fully understood, this region was replaced by a short polyglycine segment. The substantial editing and energy minimization resulted in model structures that contained a negligible amount of clashes at the expense of less ideal geometry but without a significant change in the overall MolProbity scores (see S3 File). The accuracy of the disclosed allosteric networks cannot be exactly determined; therefore, molecular dynamics simulations would be necessary to evaluate their stability.**”

**Reviewer #2:** The authors addressed satisfactorily my questions in my previous review. Based on the improvements and the scientific advance that this work represents, I recommend publication.

We thank the reviewer for the positive evaluation**.**

REFERENCES

Chirasani, V.R., M. Elferdink, M. Kral, J.S. Carter, S. Heitmann, G. Meissner, and N. Yamaguchi. 2024 Structural and functional interactions between the EF hand domain and S2-S3 loop in the type-1 ryanodine receptor ion channel. *J Biol Chem*. 300:105606.

Chirasani, V.R., K.I. Popov, G. Meissner, and N.V. Dokholyan. 2022. Mapping co-regulatory interactions among ligand-binding sites in ryanodine receptor 1. *Proteins*. 90:385-394.

Zheng, W., and H. Wen. 2020. Investigating dual Ca(2+) modulation of the ryanodine receptor 1 by molecular dynamics simulation. *Proteins*. 88:1528-1539.
